# A Comparison of Approaches to Estimate the Inbreeding Coefficient and Pairwise Relatedness Using Genomic and Pedigree Data in a Sheep Population

**DOI:** 10.1371/journal.pone.0026256

**Published:** 2011-11-09

**Authors:** Meng-Hua Li, Ismo Strandén, Timo Tiirikka, Marja-Liisa Sevón-Aimonen, Juha Kantanen

**Affiliations:** 1 Biotechnology and Food Research, MTT Agrifood Research Finland, Jokioinen, Finland; 2 Key Laboratory of Animal Ecology and Conservation Biology, Institute of Zoology, Chinese Academy of Sciences, Beijing, China; Umeå University, Sweden

## Abstract

Genome-wide SNP data provide a powerful tool to estimate pairwise relatedness among individuals and individual inbreeding coefficient. The aim of this study was to compare methods for estimating the two parameters in a Finnsheep population based on genome-wide SNPs and genealogies, separately. This study included ninety-nine Finnsheep in Finland that differed in coat colours (white, black, brown, grey, and black/white spotted) and were from a large pedigree comprising 319 119 animals. All the individuals were genotyped with the Illumina Ovine SNP50K BeadChip by the International Sheep Genomics Consortium. We identified three genetic subpopulations that corresponded approximately with the coat colours (grey, white, and black and brown) of the sheep. We detected a significant subdivision among the colour types (*F*_ST_ = 5.4%, *P*<0.05). We applied robust algorithms for the genomic estimation of individual inbreeding (*F*_SNP_) and pairwise relatedness (*Φ*_SNP_) as implemented in the programs KING and PLINK, respectively. Estimates of the two parameters from pedigrees (*F*_PED_ and *Φ*_PED_) were computed using the RelaX2 program. Values of the two parameters estimated from genomic and genealogical data were mostly consistent, in particular for the highly inbred animals (e.g. inbreeding coefficient *F*>0.0625) and pairs of closely related animals (e.g. the full- or half-sibs). Nevertheless, we also detected differences in the two parameters between the approaches, particularly with respect to the grey Finnsheep. This could be due to the smaller sample size and relative incompleteness of the pedigree for them.

We conclude that the genome-wide genomic data will provide useful information on *a per* sample or pairwise-samples basis in cases of complex genealogies or in the absence of genealogical data.

## Introduction

Genome-wide association studies (GWAS) have been widely used to identify common genetic factors that contribute to variation in complex phenotypes and influence health and disease susceptibility (see the reviews in [1]–[2]). Accurate specification of familial relationships or the integrity of pedigree information is crucial to the performance of family-based GWAS, as well as for population-based data of unknown family structure [3]. Furthermore, many linkage studies use data from small isolated populations or populations with a long tradition of marriages or matings between relatives. In these populations, the set of relationships between individuals might not be known exhaustively because genealogies can be very complex and have potentially unknown loops. As a result, a known genealogy can provide inaccurate knowledge of an individual's inbreeding coefficient [4]. High-throughput genotyping performed in GWAS represents new opportunities for complex pedigree or pedigree error detection using as many as millions of SNPs to assess the degree of relationship between a pair of individuals.

Finnsheep, the Finnish native sheep breed, has been the subject of considerable numbers of studies during recent decades. There have been studies of its reproductive and production traits (e.g. [5]–[8]), within-population genetic structure (e.g. [9]–[10]), mitochondrial maternal lineages [11] and conservation (e.g. [12]). In particular, levels of inbreeding were investigated based on pedigree records [12] and molecular markers [9]. The genetic studies thereby represent an appropriate setting for an initiative to explore the comparison of within-population estimates between pedigree and genomic information-based approaches, provided that a large set of genome-wide molecular markers are available. With the access to available pedigrees of the Finnsheep population in Finland, comprising 319 119 animals, as well as access to a novel genome-wide set of SNP markers developed for the sheep, the Illumina's Ovine SNP50K Beadchip (ISGC, International Sheep Genomics Consortium, http://www.sheephapmap.org/), comparisons between the estimates calculated using the two approaches are now realistic. Our results will also advance understanding of the Finnsheep breed in their place of origin [12] regarding their future utilisation and conservation.

In this study, we used genome-wide SNP data to characterize genetic variation in a Finnsheep population and compared the results with those derived from analysis of pedigree records. We estimated the pairwise kinship coefficient among all genotyped individuals as well as the individual inbreeding coefficient. The aim of this study was to examine the robustness of a newly developed algorithm for the relationship inference using real genome-wide SNP data and to compare the consistency between results using approaches based on genomic and pedigree information. We were also interested in elucidating the levels of genetic diversity and sub-structuring within the Finnsheep population.

## Results

### Genetic relationship and substructure within the Finnsheep population

**Figure 1:**
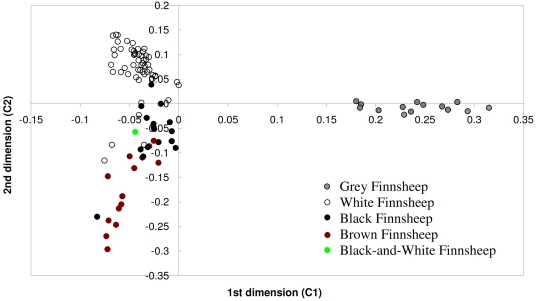
Clustering of the individual Finnsheep based on multidimensional scaling of genetic distance. The first (C1) and second (C2) dimensions are plotted.

Within-population substructure was tested using multidimensional scaling (MDS), Bayesian model-based clustering and calculation of *F*_ST_. In MDS of the identical-by-state (IBS) distance, there were three clusters that corresponded approximately with the coat colours (grey, white, and black and brown) of the sampled individuals (Fig. 1), respectively. However, the analysis was unable to differentiate between the black and brown sheep completely. The first dimension (C1) clearly separated the 14 grey individuals (C1 = 0.1802–0.315) from the others (C1 = −0.23–0), while the second dimension (C2) differentiated the white (C2 = −0.1156–0.1387) from the black and brown animals (C2 = −0.2963–0.039), with slight overlapping indicating closer genetic relationships between the two subpopulations. The analysis indicated one black/white spotted sheep to be closest to the subpopulation of black individuals (Fig. 1).

**Figure 2:**
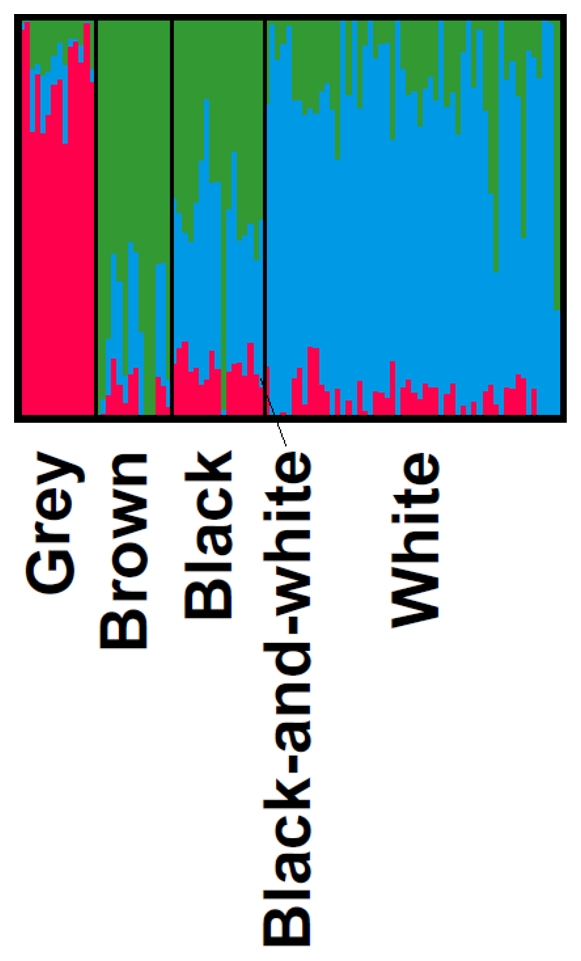
Model-based clustering of 99 Finnsheep where 3 genetic subpopulations or subclusters (*K*) were inferred. Individuals are represented in group of animals in different colours separated by vertical black lines. Each animal is represented by a single vertical line, divided into *K* colours, where *K* is the number of clusters estimated, and the coloured segment shows the individual's estimated proportion of membership to that cluster. The group of animals in different colours are given below the box plot.

Model-based clustering was further used to determine the minimum number of subpopulations (*K*) required to explain the observed total sum of within-population genetic variation. The highest average likelihood [Ln(*K*)] value and its smallest variance between replicates were obtained with *K* = 3 (data not shown), showing that *K* = 3 was the optimal number of sub-clusters for the Finnsheep population studied. Therefore, the STRUCTURE analysis found most support for three sub-clusters (or subpopulations) in the Finnsheep population, dominated by the grey, white, and black and brown Finnsheep, respectively (Fig. 2). Increasing the number of sub-clusters did not allow further differentiation. The highest genetic differentiation between pairs of subpopulations was recorded between the grey and the black and brown sheep (*F*_ST_ = 7.9%, *P*<0.05), followed by that between the grey and the white sheep (*F*_ST_ = 6.5%, *P*<0.05), while the lowest value was recorded between the white and the black and brown sheep (*F*_ST_ = 2%, *P*<0.05). Further subdivision indicated a *F*_ST_ value of 1.8% (*P*<0.05) between the black and the brown individuals. The major component of SNP variation (94.5%) occurred within the subpopulations, with only 5.4% (global *F*_ST_ = 5.4%, *P*<0.05) being diagnostic of differentiation between the three coat colour subpopulations.

### Relationship inference and individual inbreeding based on genomic data

**Figure 3:**
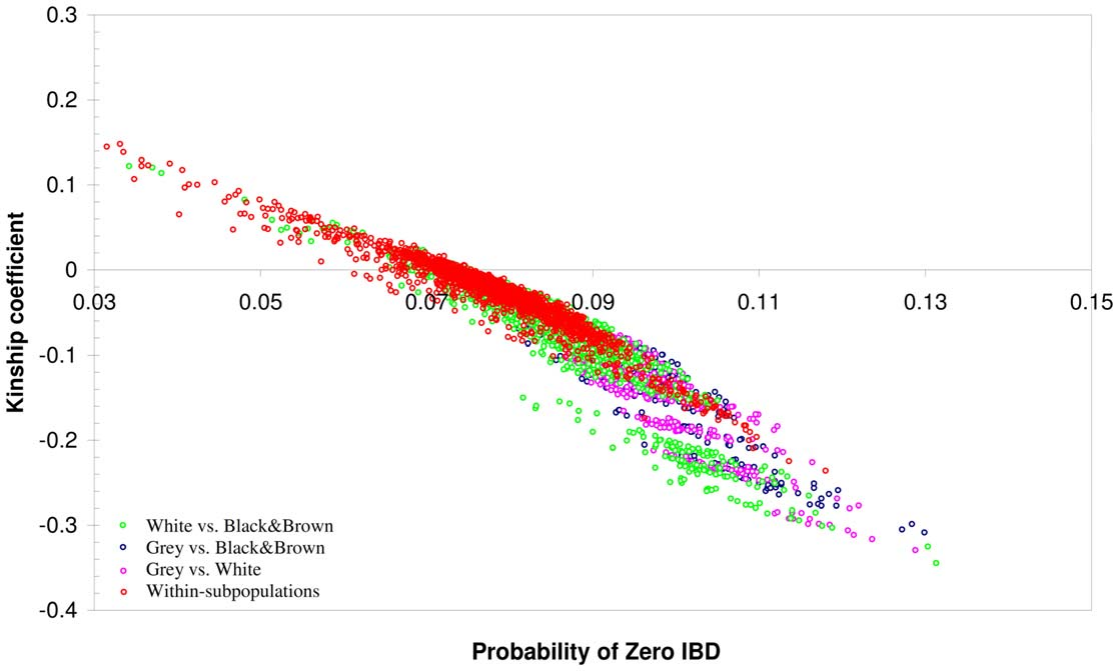
Population sub-structure in 99 SheepHapMap samples based on SNP analyses. Robust estimator of pairwise kinship coefficient (*Φ*_SNP_) as a tool for population substructure discovery. Within-population comparisons are shown in red. Other coloured dots represent comparison of individuals from distinct subpopulations.

The inferred relatedness (*Φ*_SNP_) using the KING program are illustrated in Fig. 3. We detected a high degree of consistence for the relationships between the results of genomic analyses and those indicated by pedigrees. The pairwise kinship estimator identified stratification across the pairs of distinct subpopulations, while pairs of individuals from the same subpopulation tended to constitute most of the positive inferred kinship values. All the between-group pairwise relatedness for the distantly related groups (grey *vs.* white; grey *vs.* black or brown) was negative (Fig. 3). Kinship coefficients were positive only between the pairs of animals in white *vs.* black, and those in brown *vs.* black/white spotted, which showed closer genetic relatedness between each other in general (Figs. 2,3).

**Figure 4:**
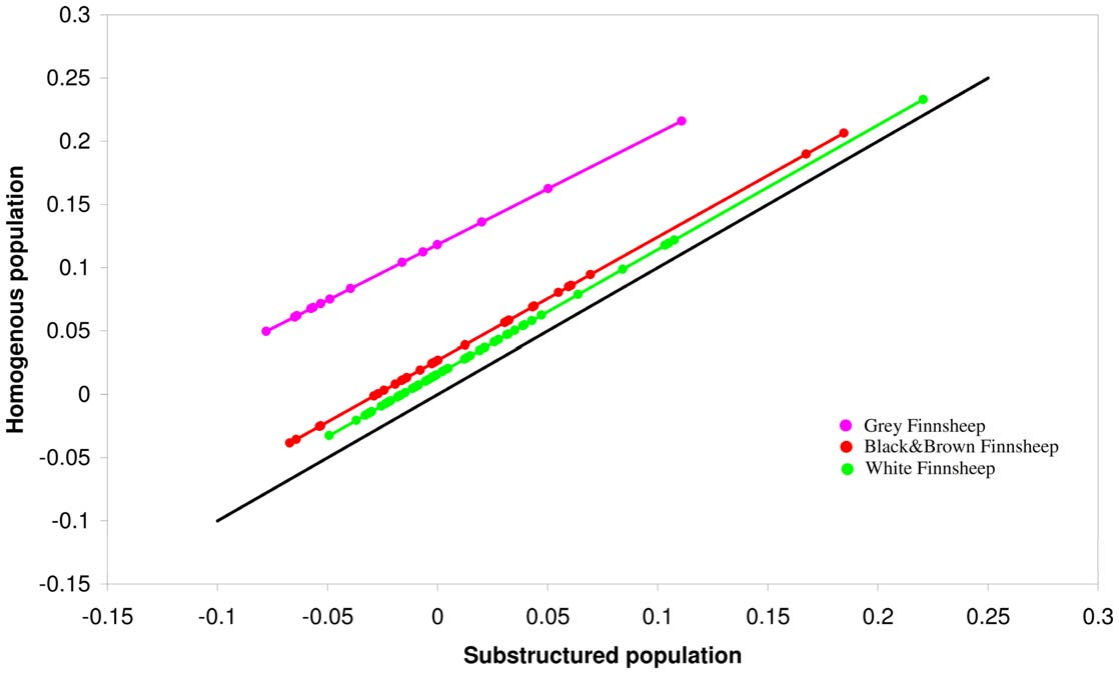
Genomic estimations of Inbreeding coefficient (*F*_SNP_) in a substructured population plotted against those of a homogeneous population. The black line follows the expectation that inbreeding coefficients are the same under the two situations.

The impact of within-population stratification on the estimation of inbreeding was reported previously in genome-wide SNP analyses (e.g. [2], [4]). In this study, the individual inbreeding coefficient (*F*_SNP_) was estimated either under an assumption of a homogeneous population including the entire sample set or in the presence of a population substructure using various subsamples according to the animals' colour (grey, white, and black and brown) as indicated by the MDS analysis. Individual inbreeding coefficients (*F*_SNP_) were comparable between the two scenarios, but the latter scenario, by incorporating population stratification, always gave systematically lower estimates (Fig. 4). The inbreeding estimates from a subsample were regressed on those from the complete population estimated under the assumption of a homogeneous population (Fig. 4). The regressions showed that the inbreeding coefficients were biased when sampling did not represent the entire sample. The bias was 0.121 (Δ_grey_ = 0.121, *n* = 14) for the grey, 0.027 (Δ_black and brown_ = 0.027, *n* = 31) for the black and brown and 0.016 for the white (Δ_white_ = 0.016, *n* = 54). This suggests that the method in an assumed homogeneous population which consists of the entire samples tends to yield inflated estimates, most likely due to the larger samples or more numerous lineages included in the analyses [13].

The mean inbreeding coefficient estimated from SNP data (*F*_SNP_) was 0.040 for the entire sample, 0.099 for the grey, 0.038 for the black and the brown, and 0.027 (results not shown) for the white Finnsheep under the assumption of a homogeneous population. Of a total of 4851 pairwise kinship coefficients estimated using the genomic data, 411 positive values were within the subpopulations and 77 were between the subpopulations. According to the relationship inference criteria based on the kinship coefficient (*Φ*) and probability of zero IBD-sharing (*π*_0_), 14 pairs were inferred to be the 1^st^ degree relatives (e.g. full-sibs), 22 pairs to be the 2^nd^ degree relatives (e.g. half-sibs), and 60 pairs to be the 3^rd^ degree relatives (e.g. first cousins; see Table S1). The estimates of *F* and *Φ* were sometimes negative but were increased to zero. As explained in [15], often such negative values can merely reflect random sampling error.

### Pedigree-based inbreeding and kinship coefficients

The pedigree completeness (*PEC*) statistic for the 99 sheep used for the pedigree analyses ranged from poor (e.g. *PEC* = 0–0.6) to excellent (*PEC* = 0.9–1). Eighteen sheep had *PEC* = 1, while the pedigree information for the majority of the samples was incomplete and 8 sheep (8.1%, 8/99) had a low level of *PEC*<0.6, ranging from 0 to 0.57.

The kinship coefficient estimated by pedigree (*Φ*_PED_) for a full-sib is always greater than or equal to 1/4 because full-sib kinship is 1/4 in an outbred population, but there can be common ancestral relatedness that increases the kinship coefficient. A kinship coefficient of *Φ* = 1/4 assumes that parents are unrelated. Accordingly, *Φ* values should be ≥1/8 or 1/16 for the 2^nd^ and 3^rd^ degree relatives, respectively (Table S1). According to the relationship inference criteria based on the kinship coefficient that was estimated from pedigree data (*Φ*_PED_), 13 pairs were inferred to be 1^st^ degree relatives (e.g. full-sibs), 36 pairs to be 2^nd^ degree relatives (e.g. half-sibs), and 125 pairs to be 3^rd^ degree relatives (e.g. first cousins; see Table S1). Of a total of 2321 positive values for the between-group kinship coefficient obtained based on the pedigree data, 278 were from the pairs in grey *vs.* white, and 261 were from the pairs in grey *vs.* black or brown.

### Comparison of inbreeding and pairwise kinship coefficients using the pedigree and genomic data

**Figure 5:**
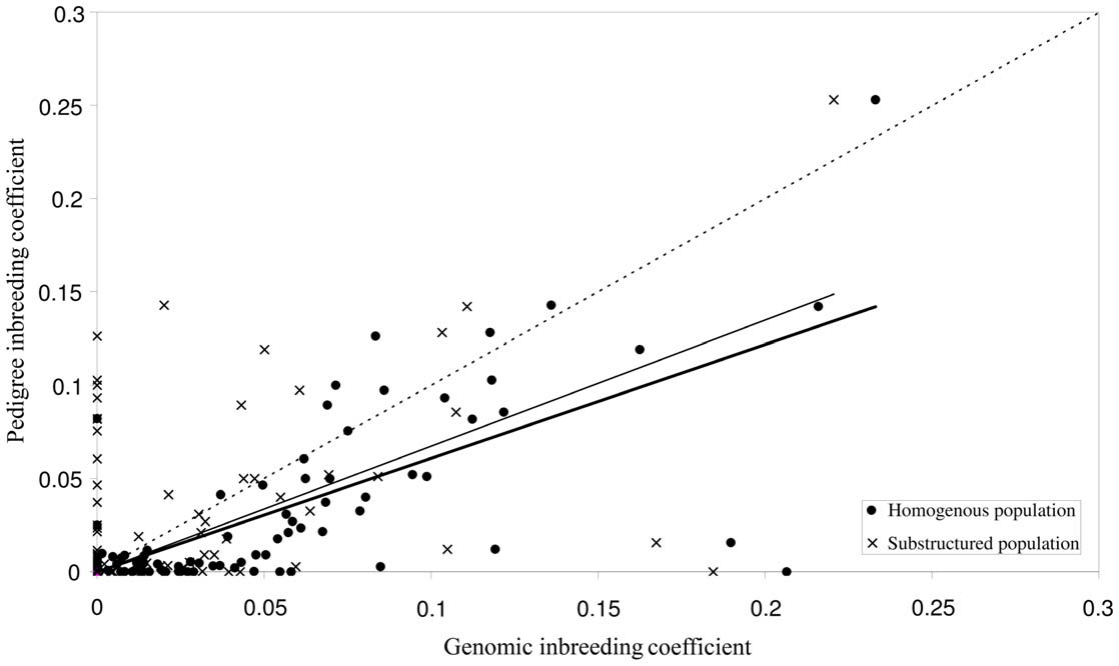
Inbreeding coefficients based on the genomic data (*F*_SNP_) assuming either a substructured or homogenous populations, plotted against inbreeding coefficients based on the pedigree data (*F*_PED_). The dash line follows the expectation that inbreeding coefficients are the same when using genomic and pedigree data; the thin-grey and bold-black lines indicate the linear regressions in assuming a substructured population or homogenous population, respectively.

The individual inbreeding coefficient estimates based on pedigree information (*F*_PED_) were compared with those calculated using SNP data (*F*_SNP_) in a homogeneous population or in the presence of population substructure (Fig. 5). The regression of *F* estimated from both the methods resulted in reasonable agreement, with an *R* ^2^ of 0.5353 and a slope of 0.6092 in the case of a homogeneous population and an *R* ^2^ of 0.4488 and a slope of 0.6740 when the population substructure was taken into account. In general, the inbreeding coefficients calculated from the pedigree information gave lower estimates than those from the genomic data. The differences varied between −0.0825 (negative value indicates that the individual estimate based on pedigree information is larger than that based on genomic data) and 0.2065 in the case of a homogeneous population, and between −0.1263 and 0.1845 when population substructure was accounted for. The proportion of animals having an inbreeding coefficient greater than 6.25%, which is the level reached by cousin mating, was 25.3% (25/99) by genomic data, 15.2% (15/99) by pedigree data and 14.1% (14/99) in both the pedigree and genomic estimations (Fig. 5).

**Figure 6:**
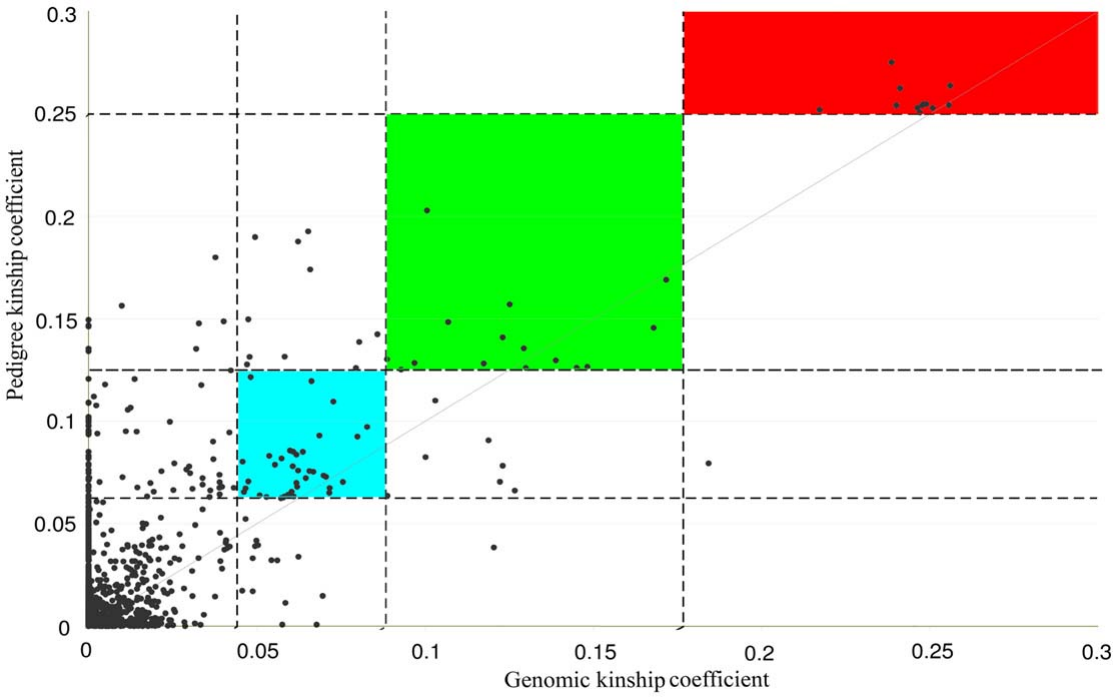
Pairwise kinship coefficients based on the genomic data (*Φ*_SNP_) in a substructured population plotted against that based on pedigree data (*Φ*_PED_). The vertical dotted lines are lower boundaries of inference criteria for the 3^rd^, 2^nd^ and 1^st^ degree relationships as in Table S1. The relationships supported by both pedigree and genetic kinship coefficients are shaded red for first degree relatives, green for second degree and turquoise for third degree relatives.

We also compared pairwise kinship coefficients between those estimated using the pedigree and genomic data (*Φ*_PED_ and *Φ*_SNP_; Fig. 6). The genomic data provided good inference for most the 1^st^ degree relatives {parents-offspring and sibling pairs, kinship coefficient *Φ* = 1/4, the inference criteria is between (1/2^5/2^, 1/2^3/2^); see Table S1 or [3]} if the kinship coefficients estimated from the pedigrees are referenced. Only one individual thought to be a 1^st^ degree relative from the SNPs was not supported by the pedigree data. Out of twenty-two 2^nd^ degree relatives (e.g. half-sibs, avuncular pairs, and grandparents-grandchild pairs) inferred by the genomic data {kinship coefficient *Φ* = 1/8, the inference criteria is between (1/2^7/2^, 1/2^5/2^); see Table S1 or [3]}, 15 were in good agreement with those estimated using the pedigrees. For the 3^rd^ degree relatives and unrelated pairs (*i.e.* the degree of relationship that lower than the 3rd degree), the kinship coefficient based on pedigree information was generally higher than that based on genomic data. The average difference for the values of pedigree- (*Φ*_PED_) and SNP-based (*Φ*_SNP_) kinship coefficients (±standard deviation; |*Φ*_PED_−*Φ*_SNP_|±S.D.) for first, second and third degree relatives and ‘non-related’ individuals are 0.021±0.0522, 0.018±0.075, 0.019±0.061 and 0.017±0.0249, respectively. Of the total 4851 [(99×98)/2] pairs of within- and between-subpopulation relationships estimated by the two approaches, 4725pairs showed a consistent degree of relationship (1^st^, 2^nd^, and 3^rd^ relatives and non-related), while 126 pairs disagreed between estimations of relationships.

## Discussion

### Comparisons between genomic and pedigree estimations

In a long-term isolated animal population such as the Finnsheep in Finland, where close relative matings are very likely, there exist complex genealogies with unknown historical loops. Therefore, the exact inbreeding coefficient (*F*) of an individual is often unknown or inaccurate when calculated using pedigree information. Here we presented an empirical example where the individual's *F* was estimated using high-density SNP genotype data from a genome-wide SNP study (*F*_SNP_), and compared the results with *F* values calculated using pedigree data (*F*_PED_). We established a reasonable correlation between the genomic estimator and the pedigree-based estimator. However, we also recorded differences in the estimates of *F* using the two approaches. There are several explanations for the differences: (*i*) incorrect pedigrees links due to all sorts of errors such as mislabelling, farmers not recording matings correctly, lambs being adopted by other mothers before they are tagged etc - *i.e.* we may have perfect knowledge of what we think the pedigrees are for these animals, but they represent incorrect links; (*ii*) the pedigree completeness, a parameter that describes the quality of available pedigree information and is of great importance in assessing inbreeding (see [12]), is low for some animals, and thus the pedigree incompleteness can cause bias in the estimation of an individual inbreeding coefficient; (*iii*) the *in silico* estimates of inbreeding are biased downward by the ascertainment bias due to being under-representative of the whole population or genealogies; and (*iv*) the requirement for a large number of samples and a denser set of SNPs to obtain accurate results.

The two approaches, based on pedigree and genomic data respectively, gave comparable estimates of pairwise kinship coefficients (*Φ*_PED_ and *Φ*_SNP_) for the 1^st^ and 2^nd^ degree relative pairs (Table S1, Fig. 6). However, we also detected a difference in the estimates of pairwise kinship coefficients between the two approaches, mostly for the unrelated and 3^rd^ degree relatives (see Fig. 6). For these pairs, the pedigree-based approach gave higher estimates for the kinship coefficient than those calculated from the genomic data. All popular algorithms for relationship inference depend on reliable estimates of allele frequencies at each SNP in a homogeneous population without stratification (e.g. [14]–[15]). Performance of the different algorithms used to classify relative pairs is affected by several factors, such as the panel of genetic markers, the underlying allele frequencies of genetic markers for different individuals and the number of individuals genotyped [3]. Since the 50K SNPs are randomly distributed across the whole sheep genome, we do not see any convincing evidence of the number and choice of genetic markers disturbing the inference of existing kinship relationships. Thus, differences in the inferred pairwise relatedness may arise from two sources. Both the existing population substructure and the relatively small size of samples investigated could lead to biased results in the genomic estimation. Nevertheless, the genome-wide SNPs will give implications in e.g. GWAS analysis in replace of pedigrees as well as in identifying (perhaps unknown) substructure within populations. The use of genomic information can be as a surrogate for pedigree data as well. In addition, many GWAS methods now adjust for unknown population structure using genetic data, and genomic selection using genome wide IBD instead of the additive relationship matrix is very widely used in livestock production. This may not be reality for the majority of Finnsheep but even with the 99 individuals genotyped at *ca.* 48K SNPs we may be able to map some “well behaved” single SNP traits.

As discussed above, we conclude that genome-wide SNPs provide more accurate information on genetic diversity of the Finnsheep than do the pedigrees. In particular, the sampling variance of SNP sharing even for full-sib pairs can be pretty huge, so accounting for true (genetic) genome sharing rather than expected (pedigree) genome sharing in linkage and genome-wide association studies can surely only improve the estimates. Nevertheless, pedigree information has been and will continue to be used in estimating population genetic parameters in the Finnsheep and other domestic animal breeds. The reasons are: (*i*) a large set of molecular markers (>10 000) at the genome-wide level has only recently become accessible; and (*ii*) the cost of comprehensive genotyping is too high. With the present approach, where only a fraction of individuals were genotyped, we were able to examine the quality of pedigrees in the Finnsheep population.

### Genomic estimation

Inbreeding coefficients calculated using genomic data (*F*_SNP_) indicated higher estimates in a homogeneous population than under population stratification. The explanation for the higher values can be that the larger sample size in a homogeneous population will always inflate the number of observed homozygotes and expected homozygotes by chance specifically for SNPs with very low MAF (minor allele frequency; see e.g. [15]). Subsequently, inbreeding coefficients are over-estimated. In addition, we noticed that an *a priori* assumption required for our robust estimator of inbreeding coefficient is linkage equilibrium (LE) among SNPs with the same underlying allele frequencies. In practice, a small proportion of SNPs deviate from the LE due to reasons including genotyping errors, recent admixture in a mixed population or removing Mendelian errors from families [3], [15]. In order to guard against potential estimation bias introduced by the departure from LE among SNPs, we estimated inbreeding in a subset of 47222 SNPs (by excluding 471 SNPs that were in LD with one or several of the others from a total of 47693 SNPs) that was adjusted to be in approximate LE. It did not substantially change the results.

The robust algorithm in the KING program performs pairwise relatedness (*Φ*_SNP_) inference using only information from the two individuals under comparison. The inference is invariant to inclusion of any additional samples and to use of different SNP panels, producing reliable results using genotypes from GWAS or from studies of rare variants alone [3]. This is the reason for the similar results for pairwise kinship coefficients (*Φ*_SNP_) in a single homogeneous population and under population stratification.

### Within-population sub-structuring

In order to examine the degree of within-population genetic sub-structuring in the Finnsheep, the distribution of SNP variation was examined as a function of membership of subpopulations with different coat colours. Within-population genetic differentiation (*F*_ST_ = 5.4%, *P*<0.05) indicated strong and significant sub-structuring among the groupings of Finnsheep of different coat colours. However, the spanning of white *vs.* black and brown sheep in the kinship coefficient estimated using genomic data indicated closer genetic similarity between these subgroups (see Fig. 3). These findings could be due to (*i*) the geographical isolation of the grey Finnsheep in the province of Kainuu in northeastern Finland, where they were discovered; (*ii*) a partly different genetic origin of the grey Finnsheep; and (*iii*) the inheritance of coat colours in the Finnsheep, where the different colour types may share the same alleles and have similar ranges of allele frequency (e.g. white *vs.* black and brown Finnsheep) at the colour genes [16]. This genetic subdivision fell into the range of the substructure (*F*_ST_ = 2.5–8.2%) reported within sheep breeds such as Dorset, Dorpers, Suffolk and Texel [17]. These values are higher than that from the results of a microsatellite-based study [9], which found that 4% of variation was explained by the colour variation in the Finnsheep. This difference observed here could be a consequence of random sampling. However, given that the microsatellites are presumed to be neutral, the higher *F*_ST_ value based on SNPs could be also due to some of the SNP markers being linked to genes affecting the economically important production traits including the coat colour and pattern, and wool quality such as like fiber diameter and its coefficient of variation, staple length and staple strength *etc*. This opens the possibility that the set markers in the SNP panel can be used for genome-wide association analysis to identify the genomic regions and mutations that underpin e.g. the coat colour trait in sheep.

The MDS and STRUCTURE analyses of the Finnsheep showed a consistent pattern of within-population genetic subdivision corresponding with the different coat colours, although with some overlapping (Fig. 1) or genetic admixture (Fig. 2) of white, black and brown animals. A similar clustering pattern of individuals within a breed was reported for the Dorpers and Merino sheep breeds, in which the populations with shared coat colour (white *vs.* black) or selection criteria (meat *vs.* wool) tended to cluster together [17]–[18]. We did not detect a geographic pattern distinguishing the Finnsheep subpopulations such as that ascribed to the genetic division between Australian Poll Dorset and American Dorset, and between African and American Dorpers [17]. The absence of geographically distinct subpopulations in the Finnsheep population studied here could be due to the limited geographic separation (*i.e.* different parts of Finland) for the samples. The differences in SNP allelic frequencies found between the three Finnsheep subpopulations could be explained on the basis of the positive assortative breeding associated with wool colour. Further tests for Hardy-Weinberg equilibrium (HWE) at the candidate loci for sheep coat colour and patterns will provide evidence of assortative mating in history of the breed. The proportion of animals with inbreeding coefficients greater than the critical level of 6.25% [12], which is the level reached by cousin mating, was 8.1% (8/99) and 14.1% (14/99) based on SNP and pedigree data, respectively (Table S1). The finding can be attributed to the effect of the avoidance of mating with the relatives within colour types that cause the low or negative inbreeding coefficients [9]. In practice, the first-cousin mating is also a critical maximum that is not exceeded when mating principles are applied on many farms in Finland (see [12]).

### Practical applications and potential caveats

The individual inbreeding coefficients (*F*) were low in the present study. Similar low levels of inbreeding in the Finnsheep population were also recorded in previous studies based on microsatellite and blood protein loci [9]–[10] as well as a comprehensive pedigree database [12]. The average inbreeding coefficient in the Finnsheep population can be considered to be below the critical level of 6.25% [12]. Thus, the estimated levels of inbreeding for the Finnsheep population, considered alone, do not justify major changes to current breeding practices. Typically, breeding on Finnsheep farms is done by mating 1 ram to 10 to 50 ewes (see [12]). Artificial insemination is not used, and there is no centralized Finnsheep breeding programme. We observed a relatively higher level of average relatedness coefficients for Finnsheep than for other sheep populations (e.g. [19]). Greater selection intensity in the breeding animals may be responsible for this observation. This finding could be also due to the fact that the number of elite breeding ewes and rams remained the same for many years. As suggested by [12], collection of samples from the pedigreed population for an animal gene bank can be based on the level of genetic relatedness as least as we knew so far. Development of germplasm cryoreserves to reintroduce genetic diversity at a later juncture could be also adopted to conserve genetic material of these animals for future utilization. Thus, with the aid of knowledge from molecular and genealogical analyses, development of viable conservation programmes, such as *in-situ* or *ex-situ* live conservation populations and germplasm cryogenic gene banks should be considered.

This study illustrates an example of genomic data being used to provide estimates of *F* with the genealogy available for comparison. However, our estimates have to be considered cautiously for three reasons. Firstly, the choice of method may affect the results since different genomic methods for estimating the inbreeding coefficient are sensitive to different parameters. For example, the maximum-likelihood method by [4] is more sensitive to rare alleles and linkage disequilibrium, while the method of [15] used in this study is more sensitive to within-population stratification, but not to linkage disequilibrium. Nevertheless, all the methods have potential advantages and drawbacks, which can be due to different underlying assumptions regarding modelling the demographic history and population stratification, as well as the uncertainness associated with the robustness of the approaches. Therefore, it is important to understand the characteristics of each method and choose the method best suited to the study. Secondly, another potential caveat of the study exists in the low sample size for some subpopulations, particularly the grey Finnsheep, which consisted of only 14 animals. Although the grey Finnsheep could have generally experienced higher inbreeding than the Finnsheep subpopulations of other colours, it would be very interesting to include at least the same or similar sizes of samples as for the white Finnsheep for comparative purposes. Thirdly, earlier simulation studies (see [20]) suggested that the use of larger marker sets to boost the statistical power may yield more precise estimates compared with studies that are based on a less dense set of markers. Thus, the next substantial advancement in the genomic estimation of individual inbreeding coefficients is likely to be based on fully sequenced sheep genomes, providing an even more precise estimate of individual genome-wide homozygosity and its distribution across the entire genome.

## Materials and Methods

### Ethics statement

The methods were approved by MTT Agrifood Research Finland, based on the regulations of the National Animal Experiment Board of Finland, Regional State Administrative Agency for Southern Finland (approval No. 81/712-94).

### Sample preparation, genotyping and quality control

Genomic DNA from a total of 99 individual Finnsheep of different coat colours (white, *n* = 54; grey, *n* = 14; black, *n* = 16, brown, *n* = 14, black/white spotted, *n* = 1) was extracted from whole blood using standard methods. DNA samples were subjected to SNP genotyping via the Illumina technology (Illumina, San Diego, California, USA), using the ovine SNP50K BeadChip as coordinated by the International Sheep Genomics Consortium (ISGC). Details on SNP discovery, design of the ovine array and genotyping procedures can be found in the ovine SNP50 HapMap dataset (http://www.sheephapmap.org/hapmap.php) and [17]. All individuals were genotyped with call rates >98% and an overall call rate of 99.99%.

Markers were excluded from the analysis if they were annotated by Illumina as having either atypical X-clustering, a nearby polymorphism, compression, intensity values only, evidence of a deletion or some combination of these assay abnormities, if their genotypes were discordant between experiments, if they showed Mendelian inconsistencies within the AgResearch International Mapping Flock created nearly a decade earlier (for details, see [21]) or animal families present within other genotypic datasets, or if the MAF was zero. A total of 49 034 SNP markers remained after the filtering. Furthermore, we excluded the SNP markers on the X, Y and unknown chromosomes (*n* = 1230, 1 and 110, respectively); thus, 47 693 SNPs on a total of 26 autosomes were maintained in the subsequent analysis.

### Pedigree data and analysis

The 99 animals selected were from a database kept by the ProAgria Association of Rural Advisory Centres in Finland. The database has maintained pedigree records for 319 119 Finnsheep individuals since 1972. The records contain information on individual identification code, sex, dam and sire identification codes, flock of origin, and birth date. Information on the pedigree data was detailed in [12].

Pedigree analysis included calculation of individual pedigree completeness (*PEC*), pedigree-based inbreeding coefficients (*F*_PED_) and pedigree-based pairwise kinship coefficients (*Φ*_PED_) using the RelaX2 program [22]. As the quality of available pedigree information is of great importance in assessing inbreeding and pairwise relatedness, a coefficient for pedigree completeness (*PEC*) was computed, and the degree of completeness of pedigree was assessed using the index proposed by [23]:
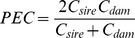
(1)

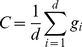
(2)In equation (1), *C*_sire_ and *C*_dam_ are contributions from the paternal and maternal lines, respectively. In equation (2), *g*_i_ is the proportion of ancestors being present in generation *i* and *d* is the total number of generations taken into account. In this study, 5 ancestral generations were considered in the calculation of *PEC* and more details were also described in [12]. Inbreeding coefficient is the probability that two alleles at a randomly chosen locus are identical by descent (IBD). The inbreeding coefficient was then calculated for all animals. The coefficient of genetic kinship (*Φ*) between animals predicts the future level of the inbreeding coefficient. We calculated pairwise coefficients of kinship between all genotyped animals.

### Analysis of pairwise relatedness and inbreeding coefficient

Given a large number of SNPs in a homogeneous sample, it is possible to calculate inbreeding coefficients (*i.e.* based on the observed *vs.* expected number of homozygous genotypes). Individual inbreeding coefficients (*F*_SNP_) estimated from genomic data were calculated using the option –het in the data set that was pruned to be in approximate linkage equilibrium using the –indep-pairwise option [window size = 50, the number of SNPs to shift the window at each step = 5, *r* ^2^ (the multiple correlation coefficient for a SNP being regressed on all other SNPs simultaneously) = 0.5] implemented in PLINK [15]. The SNP-based pairwise kinship coefficients (*Φ*_SNP_) were estimated using the KING program through the parameter –kinship. Both programs used genomic information from all genotyped animals or subsets when appropriate. Furthermore, we used the KING algorithms (KING-robust) to screen pedigree errors. Potential pedigree errors can be also viewed through graphical display, in which the inferred kinship coefficients are plotted against the estimated probability of zero-IBD. Both the kinship coefficient and the probability of zero-IBD are estimated from SNPs.

### Analysis of within-population genetic substructuring

We calculated pairwise identical-by-descent (IBD) values between each pair of individuals for all the samples. We estimated the IBD statistics by use of the average of identical-by-state (IBS) and the estimation of sample-level allele frequencies at individual SNPs assuming Hardy-Weinberg equilibrium (HWE) [3]. One hundred and forty six SNPs which showed significantly (*P*<0.01) deviation from HWE as estimated by using the option –hwe (significance level *P* = 0.01) in PLINK [15] were excluded from this analysis. Since only IBD*_ij_* = 0 rather than IBD*_ij_* = 1 or 2 between two individual indexed by *i* and *j* can result in IBS*_ij_* = 0 (*i.e.* the pair of individuals has genotypes AA and aa), the probability of zero IBD was estimated using the KING program [3].

We performed classical multidimensional scaling (MDS) on the IBS matrices of genetic distances (*D*) for all the 99 animals. The calculation of *D* is described as follows as well as earlier in [17]:
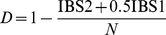
(3)where IBS1 and IBS2 are the number of loci which share either 1 or 2 alleles identical by state (IBS), respectively, and *N* is the number of loci tested. We performed the calculations using the PLINK program ([5]; available at http://pngu.mgh.harvard.edu/purcell/plink/). The extent of population substructure was further explored using STRUCTURE v 2.2 ([24]). All 99 animals were used and four replicate runs were performed for *K* = 2–6 where *K* is the number of subpopulations. In each case, the admixture model was chosen and the runs were carried out using 20 000 MCMC burn-in replications followed by a 50 000 run length. The averaged likelihood at each *K* [Ln(*K*)] and its variance between replicates was used to search for the optimal number of subpopulations (see [25]–[26]). ARLEQUIN ver. 3.11 ([27]; available at http://cmpg.unibe.ch/software/arlequin3/) was used to calculate the global and between-subpopulation genetic differentiation using the estimate of *F*_ST_.

## Supporting Information

Table S1 **Results of relationship inference based on pairwise kinship coefficient (** ***Φ*** **) and probability of zero IBD-sharing (** ***π*** **_0_) as estimated by genomic or pedigree data.**
(DOC)
