# Supplementary material for: A Comparison of Approaches to Estimate the Inbreeding Coefficient and Pairwise Relatedness Using Genomic and Pedigree Data in a Sheep Population
Source: PLoS One. 2011 Nov 9;6(11):e26256. doi: 10.1371/journal.pone.0026256 (PMC3220595; doi:10.1371/journal.pone.0026256)
Supplement: Table S1 — Results of relationship inference based on pairwise kinship coefficient ( Φ ) and probability of zero IBD-sharing ( π 0) as estimated by genomic or pedigree data. (DOC) [file pone.0026256.s001.doc]

**Table S1.** Results of relationship inference based on pairwise kinship coefficient (*Φ*) and probability of zero IBD-sharing (*π*_0_) as estimated by genomic or pedigree data

| Relationship | Examples | *Φ*^*^ | Inference Criteria | | *π*_0_^*^ | Inference Criteria^*^ | Genomic Inference ^**^ | | | Pedigree Inference^**^ |
| --- | --- | --- | --- | --- | --- | --- | --- | --- | --- | --- |
|  |  |  | Genomic^*^ | Pedigree |  |  | total pairs (proportion) | among-subpopulations | within-subpopulations |  |
| 1^st^ degree | Parent-offspring | $\frac{1}{4}$ | $\left(\frac{1}{2^{\frac{5}{2}}},\frac{1}{2^{\frac{3}{2}}}\right)$ | ≥$\frac{1}{4}$ | 0 | <0.1 | 14 (0.27%) | 0 | 14 | 13 |
|  | Full-sibs | $\frac{1}{4}$ | $\left(\frac{1}{2^{\frac{5}{2}}},\frac{1}{2^{\frac{3}{2}}}\right)$ | ≥$\frac{1}{4}$ | $\frac{1}{4}$ | (0.1, 0.365) |  |  |  |  |
| 2^nd^ degree | Half-sibs | $\frac{1}{8}$ | $\left(\frac{1}{2^{\frac{7}{2}}},\frac{1}{2^{\frac{5}{2}}}\right)$ | $\left(\frac{1}{8},\frac{1}{4}\right)$ | $\frac{1}{2}$ | (0.365, $1-\frac{1}{2^{\frac{3}{2}}}$) | 22 (0.45%) | 3 | 19 | 36 |
| 3^rd^ degree | First cousins | $\frac{1}{16}$ | $\left(\frac{1}{2^{\frac{9}{2}}},\frac{1}{2^{\frac{7}{2}}}\right)$ | $\left(\frac{1}{16},\frac{1}{8}\right)$ | $\frac{3}{4}$ | ($1-\frac{1}{2^{\frac{3}{2}}}$,$1-\frac{1}{2^{\frac{5}{2}}}$) | 60 (1.24%) | 12 | 48 | 125 |
| unrelated | - | 0 | <$\frac{1}{2^{\frac{9}{2}}}$ | <$\frac{1}{16}$ | 1 | >$1-\frac{1}{2^{\frac{5}{2}}}$ | 4755 (98.02%) | 2849 | 1906 | 4677 |

^*^ See [Manichaikul A](http://www.ncbi.nlm.nih.gov/pubmed?term="Manichaikul A"%5BAuthor%5D), [Mychaleckyj JC](http://www.ncbi.nlm.nih.gov/pubmed?term="Mychaleckyj JC"%5BAuthor%5D), [Rich SS](http://www.ncbi.nlm.nih.gov/pubmed?term="Rich SS"%5BAuthor%5D), [Daly K](http://www.ncbi.nlm.nih.gov/pubmed?term="Daly K"%5BAuthor%5D), [Sale M](http://www.ncbi.nlm.nih.gov/pubmed?term="Sale M"%5BAuthor%5D), et al. (2010) Robust relationship inference in genome-wide association studies. [Bioinformatics](javascript:AL_get(this, 'jour', 'Bioinformatics.');) 26:2867-2873.

^**^ Number of pairs
